# Puzzle based teaching versus traditional instruction in electrocardiogram interpretation for medical students – a pilot study

**DOI:** 10.1186/1472-6920-9-4

**Published:** 2009-01-13

**Authors:** Jack Rubinstein, Abhijeet Dhoble, Gary Ferenchick

**Affiliations:** 1 Division of Cardiology, Department of Internal Medicine, Michigan State University, East Lansing, Michigan, USA; 2 Department of Internal Medicine, Michigan State University, East Lansing, Michigan, USA

## Abstract

**Background:**

Most medical professionals are expected to possess basic electrocardiogram (EKG) interpretation skills. But, published data suggests that residents' and physicians' EKG interpretation skills are suboptimal. Learning styles differ among medical students; individualization of teaching methods has been shown to be viable and may result in improved learning. Puzzles have been shown to facilitate learning in a relaxed environment. The objective of this study was to assess efficacy of teaching puzzle in EKG interpretation skills among medical students.

**Methods:**

This is a reader blinded crossover trial. Third year medical students from College of Human Medicine, Michigan State University participated in this study. Two groups (n = 9) received two traditional EKG interpretation skills lectures followed by a standardized exam and two extra sessions with the teaching puzzle and a different exam. Two other groups (n = 6) received identical courses and exams with the puzzle session first followed by the traditional teaching. EKG interpretation scores on final test were used as main outcome measure.

**Results:**

The average score after only traditional teaching was 4.07 ± 2.08 while after only the puzzle session was 4.04 ± 2.36 (p = 0.97). The average improvement after the traditional session was followed up with a puzzle session was 2.53 ± 1.94 while the average improvement after the puzzle session was followed with the traditional session was 2.08 ± 1.73 (p = 0.67). The final EKG exam score for this cohort (n = 15) was 84.1 compared to 86.6 (p = 0.22) for a comparable sample of medical students (n = 15) at a different campus.

**Conclusion:**

Teaching EKG interpretation with puzzles is comparable to traditional teaching and may be particularly useful for certain subgroups of students. Puzzle session are more interactive and relaxing, and warrant further investigations on larger scale.

## Background

The electrocardiogram (EKG) is a basic and commonly used diagnostic test in modern medicine. Most medical professionals are expected to possess basic EKG interpretation skills and as such it has become part of the curriculum for medical students undergoing their internal medicine rotations and training [1].

A review of the recent literature in the area of EKG interpretation has found the reading skills of interns [2], residents [3] and even attending physicians [4] in several specialties including internal medicine [5], family practice [6] and emergency medicine [7] are suboptimal and potentially deleterious to patient care [8]. Furthermore, there are very few studies that have explored ways to improve EKG interpretation skills [9].

Most of these publications present varied time frames and teaching techniques and have had only marginal and non-sustained improvement in interpretation skills. Even studies that have looked into the addition of computer generated diagnosis and neural networks [10] have shown no improvement [11], minor improvement [12] and in some cases worsening [13] in diagnostic accuracy. Furthermore, the outcomes in these studies are not standardized and their results are difficult to implement [14].

The most current and exhaustive review on the subject states that there is no current evidence based criteria that improves and maintains EKG interpretation skills [15] and that further research is needed to clarify and improve the currently available methods [16], or devise a new method.

Traditional medical teaching aims to teach students by explaining the underlying mechanisms of disease through basic science learning, followed by disease categories and finally attempts to bring the learning together by demonstrating these mechanisms in patients [17]. Alternatively, problem based learning (PBL) aims to teach the basic and clinical concepts of disease through the course of solving a clinical problem [18]. Interestingly, neither method has been shown to improve the educational performance or EKG interpretation skills. It has been suggested that this is due to a lack of deeper understanding of the material presented and hence the prerequisite for successful transfer of medical information to relevant conceptual knowledge may be missing [19].

We have developed a puzzle (patent pending MSU ID 07-120) that attempts to improve the students overall understanding of the EKG and subsequently improve their interpretation skills. This tool is devised to encourage deductive reasoning and critical thinking instead of passive memorization of the material. The aim of the puzzle is to make the students active learners, and utilize their skills outside of the classroom to complement their knowledge base.

Similar approaches, such as card games [20] and simulations [21], have been shown to enhance students' level of understanding and ability to apply and synthesize material. This kind of self directed learning encourages the development of individualized and deeper understanding, and may produce physicians better prepared for lifelong education. PBL is a very popular instructional strategy and is particularly suitable for professional education [22]. PBL exploit the merits of learner centered approach, and promotes deep, rather than surface learning [22,23]. The traditional teaching approach is perceived as stressful by many students, and there is a need for novel methods to be used to encourage interactive teaching [23,24]. Moreover, doctors with greater stress and emotional exhaustion during training have shown to be more disorganized in their approach in their practice, less satisfied with medicine as a career, and perceive their workplace climate as stressful environment with high workload [24].

### Objective

We sought to compare EKG puzzle sessions with traditional teaching sessions in a group of medical students undergoing their medicine clerkship with standardized EKG tests. Furthermore, we evaluated their perceived stress response to both sessions.

## Methods

### Study population

Four groups of third year medical students (n = 15) undergoing their internal medicine clerkship rotation were offered the opportunity to participate in this study. The Institutional Review Board of Michigan State University approved the study and all participants gave written and informed consent to participate. All students participated and underwent their standard two-month clerkship and teaching with the added puzzle session. We also compared their final EKG scores with the scores of representative medical students (n = 15) from a different campus of the same school who underwent traditional teaching during same time period.

### Study design

**Figure 1:**
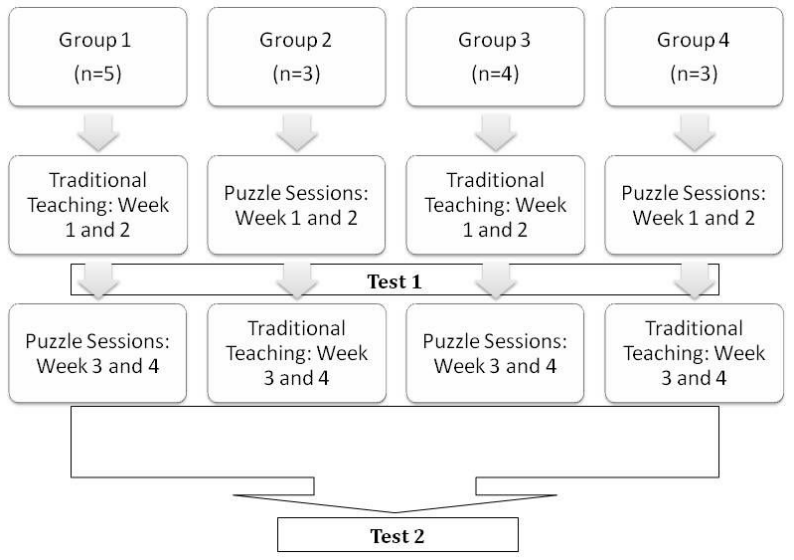
Lecture schedule for students.

This was a reader blinded crossover trial. All students were given two separate 2 hour-long standard EKG interpretation lectures and were also given two separate 2 hour-long puzzle sessions. Traditional 2-hour lectures were given at the beginning of the rotation for groups one and three, followed by the puzzle classes. For the second and fourth groups, the puzzle classes were given first, followed by the traditional lectures (figure 1). The same standardized test of five EKGs was administered after the first teaching sessions and subsequently five more were given at the end of the second sessions. For each test there was a 20-minute maximum time allowance, the students were under constant supervision and no assistance was provided.

Prior to the course students were asked general information about their age, sex, prior exposure to EKG training (1 = none, 5 = significant) and how important they consider to be EKG interpretation in their career (1 = none to 5 = significant). At the conclusion of the final exam feedback was asked from the students by asking the following questions: 1. How useful was the teaching method (1 = not useful to 5 = very useful), 2. Was the method stressful (yes/no), 3. fun (yes/no), 4. time well spent (yes/no), 5. engaging (yes/no).

### The puzzle

**Figure 2:**
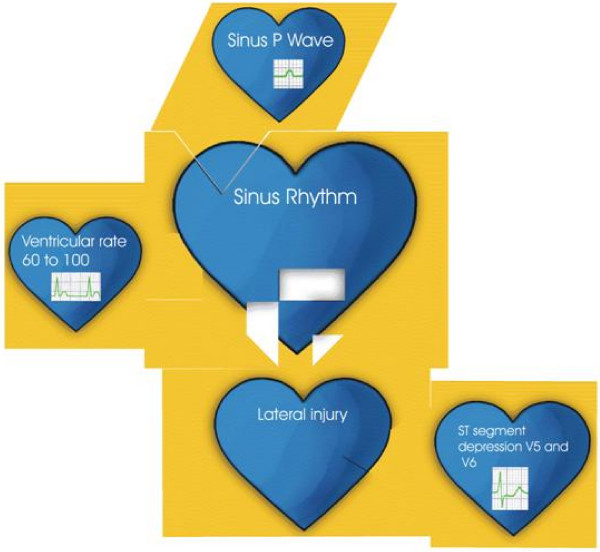
Example of complete diagnosis of sinus rhythm and lateral injury.

This puzzle consists of 18 basic diagnostic pieces that include the most common basic EKG diagnosis such as "sinus rhythm", "atrial tachycardia" and "atrial flutter" to name a few; these pieces can match with 34 accessory pieces with common secondary EKG diagnosis such as "left bundle branch block", "first degree AV block" and "hyperkalemia". These basic and accessory pieces are interchangeable only when they make diagnostic sense; for example "first degree AV block" can fit with "sinus rhythm" but not with "junctional rhythm". There are also 47 add-on pieces that demonstrate the electrocardiographic findings associated with the basic and accessory pieces (i.e. a sinus p wave for sinus rhythm and peaked T waves for hyperkalemia) (figure 2). All pieces have a brief explanation of the finding and EKG criteria on the back.

### Outcome measure and scoring

Each test EKG was graded as per the university's protocol with a maximum of 10 points; five points for the correct diagnosis, one point each for rate, rhythm, axis, understanding of need for immediate attention and 0.5 points given for correctly measuring PR and QRS durations.

Each test included five EKGs with both sets standardized and consistent with each other. They were structured to test students capacity to diagnose sinus rhythm (n = 2), bundle branch blocks (n = 1), myocardial infarction (n = 1), and rhythm abnormalities (n = 1). The key for the exam was established by two senior cardiology fellows and was subsequently graded by a blinded physician.

### Data analysis

Analysis was performed with Graph-Pad In-Stat 3.06 (GraphPad Software, San Diego, CA, USA). The groups were divided by their initial instruction method and two separate group scores were analyzed with the results presented as mean and standard deviation (SD) in parenthesis. The difference among these groups and between the initial and subsequent exam was also analyzed for mean and SD, and subsequently subjected to a two-sample Student's *t*-test for unequal variance to calculate p-values. A p-value < 0.05 was considered statistically significant.

## Results

A total of 15 students participated in the course and 14 completed both the clerkship and the exams (93%). The average age was 29 years, and all considered EKG interpretation to be an important (30%) or very important (70%) skill to have. Most had some self reported EKG exposure prior to the course, with 11/13 (84%) using books and 5/13 (39%) during clinical rounds (table 1). Nine students underwent traditional teaching first followed by the puzzle session and six underwent the puzzle teaching method initially followed by traditional sessions. The average score after only the traditional teaching was 4.07 ± 2.08 while after only the puzzle session was 4.04 ± 2.36 (p = 0.97).

**Table 1:** Baseline characteristics of students

	Traditional	Puzzle	p value
Male (%)	22%	16%	0.32
Age (years)	29	29	0.9
Average experience *	2	2	0.14
Importance of EKG interpretation in career *	5	5	0.86
Prior clinical (%)	28%	50%	0.47
Prior books (%)	100%	66%	0.03

*. 1 = none, 2 = minimal, 3 = some, 4 = a lot, 5 = significant

**Figure 3:**
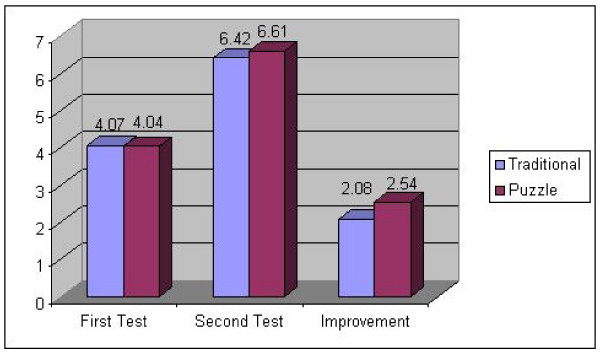
**Average initial, final score and improvement by teaching method**.

The average improvement after the traditional session followed up with a puzzle session was 2.53 ± 1.94 while the average improvement after the puzzle session was followed with the traditional session was 2.08 ± 1.73 (p = 0.67) (figure 3). The average final score after the full rotation and both teaching techniques was 6.54 ± 1.69.

There were five (55%) students that had an average score of less than five after the traditional teaching sessions. These students subsequently had an average improvement of 3.64 ± 1.03 after the puzzle session and their mean scores were not significantly different from those students that had an average score of greater than five after the traditional teaching. Three (50%) students had an average score less than 5 after their initial puzzle sessions and their scores improved after the traditional session by an average of 3.01 ± 1.48 (p = 0.28).

After just the traditional teaching, 3/9 (33%) were able to identify normal sinus rhythm, 1/9 (11%) atrial fibrillation and 4/9 (44%) acute myocardial infarction, while after just the puzzle teaching 2/6 (33%) were able to identify normal sinus rhythm, 2/6 (33%) atrial fibrillation and 3/6 (50%) acute myocardial infarction. At the final test examination after both sessions, 78% (11/14) of students were able to correctly identify normal sinus rhythm, 57% (8/14) were able to correctly identify left bundle branch block, 64% (9/14) acute myocardial infarction and 57% (8/14) atrial flutter (table 2).

**Table 2:** Diagnostic accuracy after first session and at final exam

EKG interpretation	After Traditional	After Puzzle	Final Test
Normal	33%	33%	78%
Left Bundle Branch Block	NT	NT	57%
Acute Infarction	44%	50%	64%
Third Degree AV block	33%	17%	NT
Atrial Fibrillation/Flutter	11%	33%	57%

NT = Not tested

All students considered the traditional session's time well spent while 69% had the same opinion of the puzzle teaching. Furthermore, 46% considered the traditional method stressful while only 17% shared that opinion of the puzzle sessions. There was no difference in scores after the traditional sessions in those that considered the method stressful (4.1 ± 2.79) compared to those that did not (4.05 ± 1.1) (p = 0.48) or in those that considered the puzzle session stressful (4.7 ± 4.24) and those that did not (3.7 ± 1.68) (p = 0.39).

The final university EKG exam scores of this group of student from our campus (n = 15) to medical students (n = 15) was comparable to that of a similar group of students from a different campus who underwent a similar amount of traditional teaching sessions and were given the same exam (84.1 ± 6.25 vs. 86.6 ± 5.10, p = 0.22).

## Discussion

We have confirmed what most of the trials that we looked at stated in regards to the difficulty involved in teaching adequate EKG interpretation skills to medical students [2-9]. Even with the combined teaching modality, their average score was only 6.5, and a significant number had difficulty identifying common electrocardiographic diagnoses.

In this population of medical students, we did not detect any statistical improvement in EKG interpretation skills after either the traditional or the puzzle teaching. When we compared this group of students with a different contemporaneous group of students the scores were also comparable. However, a statistically significant difference would be difficult to find in light of the small sample size.

The puzzle sessions was helpful in the subgroup of students that did poorly after the traditional teaching showing an average improvement of 3.64 ± 1.03. This group included approximately half of the student population that was initially exposed to traditional teaching and potentially included students that did not recognize EKG patterns as easily as their colleagues but were able to obtain a correct EKG diagnosis through other mental mechanism. Unfortunately, we were not able to identify this subgroup of students by their perceived level of stress.

PBL [25] and self directed learning [26] have both been shown to improve outcomes in different teaching environments. The puzzle sessions incorporated several key points from these theories including letting the students take the initiative in the learning process, permitting the student to identify his and her own weaknesses, and permitting the individual to identify the diagnostic problem and look for an appropriate solution (or piece). We believe that part of the improvements seen in these cases were due to the application of these theories in a practical environment [23]. Clearly, more research on a larger scale is required to further investigate these links and potential benefits.

The students that scored poorly after the puzzle session showed improvement after the traditional teaching, but they did not reach the same level of competency in comparison to the other students (5.8 vs. 7.35). This suggests that the students who underwent puzzle sessions first set their own learning goals, and generated a challenging educational environment before taking traditional teaching courses. Again, this emphasizes that non-traditional teaching is an effective way of delivering medical education in a coherent way and offers advantages over traditional teaching methods [20,21]. Hence, individualizing EKG teaching with various methods available to the teacher may result in improved EKG interpretation skills.

All students thought that the traditional teaching method was useful and that the time well spent, even though it was considered to be stressful by some (46%). The puzzle session was considered useful by a significant number of students (69%) and was widely regarded as less stressful (17%). As pointed out earlier, increased stress level during physicians' training adversely affects their perceived level of job satisfaction and may decrease their capacity for deeper learning and understanding of complex ideas [24]. Thus, the relaxed atmosphere of the puzzle session may have nurtured self directed problem based learning, and potentially improved the interpretation skills.

It has been shown that EKG interpretation accuracy improves with advancing years of training [7] and with further classes and lessons [5] and it is possible that the extra teaching with puzzle played a role in improving the final EKG scores. The puzzle sessions trended towards better initial scores and higher improvement in scores than traditional teaching but no trend proved to be statistically significant.

### Strengths and weakness of the study

The limitations of this study include a small sample size from only one class of students from same university. Furthermore, it included exclusively medical students and was based on an experimental prototype of the teaching puzzle. We will be improving the initial prototype with changes to the design for clarity and correction of minor technical errors that were found during the testing. We will also apply this technique to other areas in medical training (student and registered nurses, family and internal medicine residents, etc.) and in other medical specialties to attempt to identify the groups that may benefit the most.

## Conclusion

We have demonstrated that this different and novel teaching technique with puzzles is at least as useful as traditional EKG teaching and is more interactive and learner centered. It may be particularly useful for students that may learn more efficiently in relaxed environments that are more learner-centered.

## Ethical approval

This project was approved by the Institutional Review Board of Michigan State University.

## Consent

An informed consent was obtained from each participant in the study.

## Competing interests

Primary author is in the process of patenting the puzzle. The abstract with preliminary data was presented at Lansing Research Day 2008 at Lansing, Michigan and the 2008 national scientific meeting of the American College of Physicians in Washington, D.C. The other authors declare that they have no competing interests.

## Authors' contributions

JR was involved in developing puzzle, conducting puzzle sessions, collecting data, and conceptualizing framework of the manuscript. AD was involved in revising manuscript critically, and providing input regarding results, and statistical analysis. GF was involved in formulating the standardized tests and methodology. All authors read and approved the final manuscript.

## Pre-publication history

The pre-publication history for this paper can be accessed here:


